# LaIT6: A Novel Insect-Selective K^+^-Channel Toxin from *Liocheles australasiae* Scorpion Venom

**DOI:** 10.3390/molecules30163346

**Published:** 2025-08-11

**Authors:** Konoka Kumagai, Takumi Kishimoto, Kathleen Carleer, Nana Butatsu, Tsubasa Teramoto, Naoya Mitani, Jan Tytgat, Yoshiaki Nakagawa, Masahiro Miyashita

**Affiliations:** 1 Graduate School of Agriculture, Kyoto University, Kyoto 606-8502, Japan; 2 Toxicology and Pharmacology, University of Leuven, 3000 Leuven, Belgium; kathleen.carleer@kuleuven.be (K.C.);

**Keywords:** scorpion venom, insecticidal peptide, potassium channel, chemical synthesis, selective action

## Abstract

Scorpion venom contains various insecticidal peptides. Previously, through transcriptome analysis of the venom gland of *Liocheles australasiae*, we identified precursor sequences of several peptides that share sequences similar to those acting on K^+^ channels. In this study, we chemically synthesized five of the peptides which were found in the venom and evaluated their insecticidal activity against crickets. This revealed that one of the peptides, named LaIT6, exhibited significant insecticidal activity without mammalian toxicity. To identify amino acid residues important for the insecticidal activity of LaIT6, nine analogs were synthesized mainly by substituting acidic, basic, and aromatic residues with alanine. This revealed that two basic residues and an aromatic residue in the C-terminal region are important for the activity. This characteristic of structure-activity relationships, known as a functional dyad, is commonly observed in peptides that act on K^+^ channels, suggesting that the action target of LaIT6 is K^+^ channels. As expected, LaIT6 showed significant inhibitory activity against insect K^+^ channels. Since no activity against human K^+^ channels was observed, we concluded that the selectivity of LaIT6 is determined by differences in the action on K^+^ channels between insects and mammals.

## 1. Introduction

Scorpions use venom to capture prey and defend against predators [1]. Therefore, scorpion venom contains components that exhibit insecticidal activity and mammalian toxicity [2,3,4]. These components mainly act on the nervous system, particularly ion channels (Na^+^, K^+^, Ca^2+^), to immediately stop the movement of prey or to cause sharp pain to predators [5]. The venom components responsible for these functions are peptides, most of which are cross-linked by multiple disulfide bonds. In addition, some of these peptides are known to show highly selective toxicity against insects without affecting mammals, which provides an opportunity for biopesticide application [6]. Moreover, scorpion toxins with high action selectivity are not only useful as tools for studying ion channel functions [7], but also provide insights into the mechanisms underlying their functional and structural diversification [8].

Among scorpion peptides, those acting on voltage-gated Na^+^ channels (NaTx) have been identified and studied extensively for decades [9,10,11]. In general, NaTx peptides consist of 60–72 residues and are cross-linked by four disulfide bonds to form a cystine-stabilized α-helix and β-sheet (CS α/β) motif. NaTx peptides are classified into two types (α- and β-toxins) based on differences in their mode of action on Na^+^ channels. They are further classified into two types based on differences in selectivity between insects and mammals. However, since NaTx peptides are primarily present in the venom of the Buthidae family, it can be surmised that the insecticidal activity in the venom of the non-Buthidae family is caused by other types of toxins.

Scorpion venom also contains a large number of peptides that act on K^+^ channels (KTx) [12,13]. KTx peptides are classified into seven families based on their structures and functions (α, β, γ, κ, δ, λ, and ε). Many α-KTx peptides, which consist of 23–43 residues to form a CS α/β motif with three or four disulfide bonds, have been reported to act on K^+^ channels at pico- to nanomolar concentrations [14,15]. However, most of their activities have been evaluated against mammalian K^+^ channels, with very few showing insect-selective toxicity. Given that one of the main purposes of scorpion venom is to capture insects as prey, it is reasonable to expect that unknown insecticidal peptides could be found among the abundance of KTx peptides in the venom. In previous studies, we demonstrated that several β-KTx peptides (LaIT2 and LaIT3) in the venom of the *Liocheles australasiae* scorpion contribute to its insecticidal activity [16,17]. Furthermore, we recently discovered a novel insecticidal κ-KTx peptide (LaIT5) through transcriptome analysis of the *L. australasiae* venom gland, although its mode of action remains unknown [18]. These findings suggest that other KTx peptides in the venom may also have insecticidal activity. Therefore, in this study, we searched for novel insecticidal α-KTx peptides present in *L. australasiae* venom.

## 2. Results

### 2.1. Identification of α-KTx Peptides in *L. australasiae* Venom

**Figure 1:**
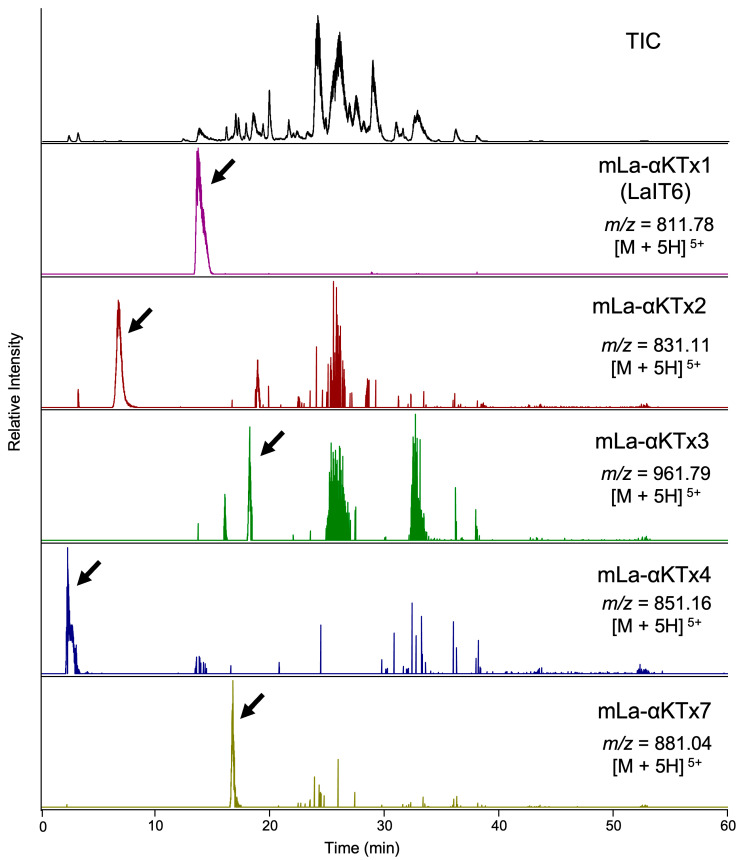
Detection of α-KTx peptides in *L. australasiae* venom by high-resolution LC/MS analysis. The x-axis represents the retention time (min), and the y-axis indicates the relative ion intensity. The top trace shows the total ion current (TIC) chromatogram, and the traces below show the extracted chromatograms of protonated molecules ([M + 5H]^5+^) for each α-KTx peptide. Arrows indicate the peaks of detected α-KTx peptides.

We first examined the presence of α-KTx peptides in *L. australasiae* venom. The precursor sequences of these peptides were previously deduced through de novo transcriptome analysis of the venom gland [19]. Mature sequences were estimated by removing the signal sequence and propeptide regions from each precursor sequence (Table 1). Peptides with calculated molecular masses of the estimated mature sequences were searched from the mass data obtained through high-resolution LC/MS analysis of the venom. As a result, five components with molecular masses consistent with those of estimated α-KTx peptides were detected in the venom (Figure 1). These peptides were chemically synthesized using the Fmoc solid-phase method, followed by the folding reaction using a redox buffer (Figure S1). Comparison of retention times in LC/MS analysis between synthetic and venom-derived peptides revealed that they are identical, confirming their presence in the venom (Figure S2).

### 2.2. Bioactivity Evaluation

Five synthetic α-KTx peptides were evaluated for insecticidal activity against crickets. Only mLa-αKTx1 showed insecticidal activity, with a dose required to induce death in half of the animals (LD_50_) of 16 nmol/g (Table 2). The insecticidal activity of this peptide was lower than that of LaIT1 (LD_50_ of 5.2 nmol/g) [20], the most potent insecticidal peptide in *L. australasiae* venom, but was comparable to those of LaIT3 (LD_50_ of 12 nmol/g) and LaIT5 (LD_50_ of 10 nmol/g) [18,21]. Therefore, we named it LaIT6, considering the number of insecticidal toxins found in *L. australasiae* venom.

We further examined the insecticidal activity of LaIT6 against other insect species. LaIT6 also exhibited significant insecticidal activity against houseflies (*Musca domestica*), with more than half of the tested insects dying after injection of 16 nmol/g. However, we found that it was not toxic to silkworms (*Bombyx mori*) or cockroaches (*Periplaneta americana*), even at injection doses of 50 nmol/g and 25 nmol/g, respectively. This suggests that LaIT6 has insect-order-specific toxicity, particularly against orthopterans and dipterans. We also evaluated the mammalian toxicity of LaIT6 by injecting intracerebrally into mice. However, we did not observe any toxic symptoms, even at an injection dose of 50 nmol/kg. In comparison, Pi1, an α-KTx peptide with mammalian toxicity, has an LD_50_ of 2.6 nmol/kg [22]. Therefore, it can be concluded that the action of LaIT6 is highly insect-selective without affecting mammals.

### 2.3. Structure–Activity Relationship

The acidic, basic, and aromatic amino acid residues in α-KTx peptides are known to be important for their interaction with ion channels, particularly K^+^ channels [23,24]. To evaluate their importance for the insecticidal activity of LaIT6, nine analogs were synthesized by substituting these residues with alanine or by converting the C-terminal amide group to a carboxyl group (Figure S3). Evaluation of the insecticidal activity against crickets showed a significant decrease in activity when each of the three residues (K28, K35, and Y37) in the C-terminal region was substituted with alanine (Table 3). On the other hand, no or only a slight change in activity was observed in other analogs.

**Figure 2:**
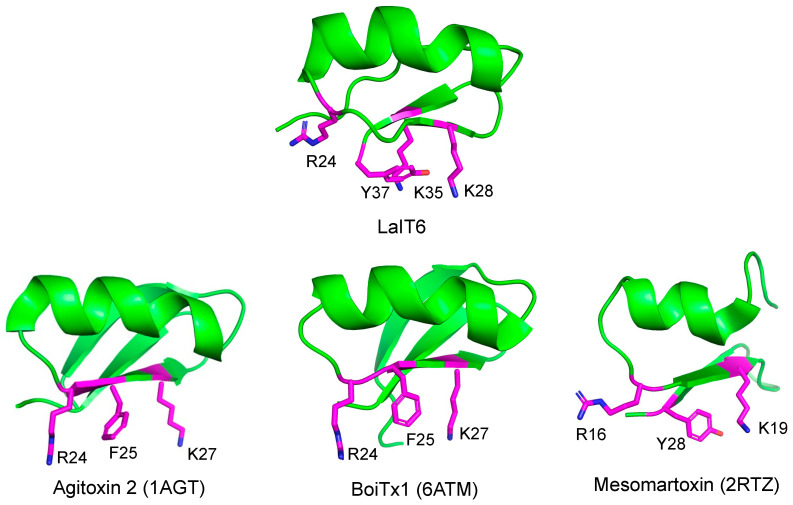
Comparison of the spatial position of residues important for the insecticidal activity of LaIT6 with those of other insect-specific α-KTx peptides. The three-dimensional structure of LaIT6 was estimated using AlphaFold 3. PDB IDs of agitoxin 2, BoiTx1, and mesomartoxin are indicated in parentheses.

The three-dimensional structure of LaIT6 was predicted using AlphaFold 3 [25]. The structure showed high reliability with a pLDDT score > 90, except for the N-terminal four residues, indicating that it is a highly reliable model (Figure S4). In this structure, the three residues (K28, K35, and Y37) important for insecticidal activity are closely located on the C-terminal β-sheet structure (Figure 2). This characteristic of structure-activity relationships is commonly observed in peptides that act on K^+^ channels, in which the combination of basic and aromatic amino acid residues is critical for interaction with the pore region of K^+^ channels as a functional dyad [23]. This strongly suggests that LaIT6 targets insect K^+^ channels.

### 2.4. Effect of LaIT6 on K^+^ Channels

**Figure 3:**
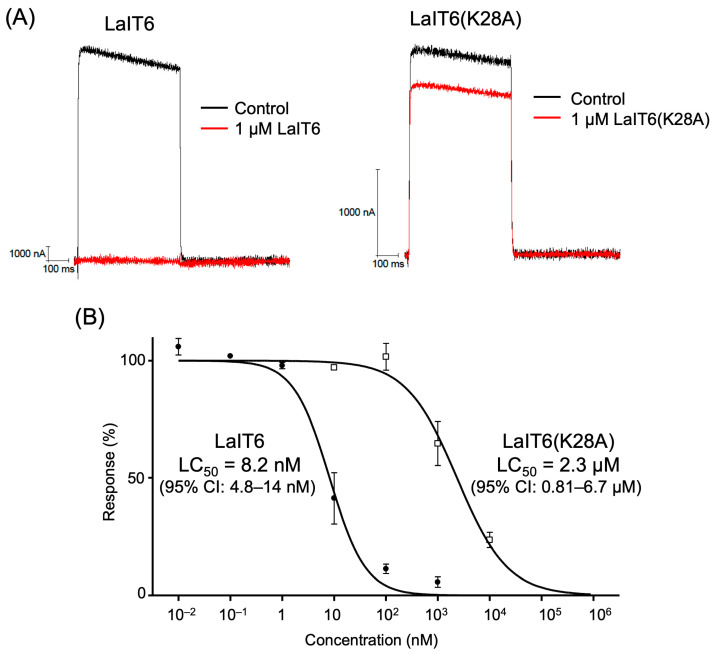
Inhibitory activity of LaIT6 and LaIT6(K28A) against the Shaker-IR K^+^ channel. (**A**) Representative currents of the Shaker-IR K^+^ channel in the presence and absence of 1 µM LaIT6 and LaIT6(K28A). (**B**) Concentration dependence of inhibitory activity of LaIT6 (closed circle) and LaIT6(K28A) (open square) toward Shaker-IR K^+^ channels. The *x*-axis represents the log-transformed concentration (nM), and the y-axis shows the percentage of currents relative to the control. Each data point shown is the mean ± S.D. (*n* ≥ 3). The IC_50_ values and their 95% confidence intervals (CI) for LaIT6 and LaIT6(K28A) are shown in the graph.

The effects of LaIT6 on the insect K^+^ channel (*Drosophila* Shaker-IR) expressed in *Xenopus* oocytes were electrophysiologically evaluated (Figure 3). The result indicated that LaIT6 has significant inhibitory activity against the Shaker-IR K^+^ channel, with a half maximal inhibitory concentration (IC_50_) of 8.2 nM. Next, we evaluated the inhibitory activity of LaIT6(K28A), an analog without insecticidal activity, against the Shaker-IR K^+^ channel. In this analog, the 28th Lys residue, which is likely the most important due to forming the functional dyad with the C-terminal Tyr residue, was substituted with Ala. It was expected that its inhibitory activity against the Shaker-IR K^+^ channel would be greatly reduced. As expected, the IC_50_ value of LaIT6(K28A) was determined to be 2.3 µM, which is approximately 300 times higher than that of wild-type LaIT6. We then examined the activity of LaIT6 against human K^+^ channels (hKv1.1 and hKv1.3), but it did not exhibit inhibitory activity against either of the K^+^ channels, even at a concentration of 1 µM. LaIT6(K28A) also showed no inhibitory activity against hKv1.1 at a concentration of 1 µM.

## 3. Discussion

In this study, we chemically synthesized five α-KTx peptides with unknown functions found in the venom of *L. australasiae* and measured their insecticidal activity. We discovered that one of the synthetic α-KTx peptides, which we named LaIT6, exhibited significant insecticidal activity without any toxicity to mammals. Furthermore, the structure-activity relationship study strongly suggested that LaIT6 targets K^+^ channels. Electrophysiological experiments revealed that LaIT6 acts on the insect K^+^ channel (Shaker-IR) at low nanomolar concentrations, whereas it showed no effect on human K^+^ channels (hKv1.1 and hKv1.3), even at micromolar concentrations.

**Figure 4:**
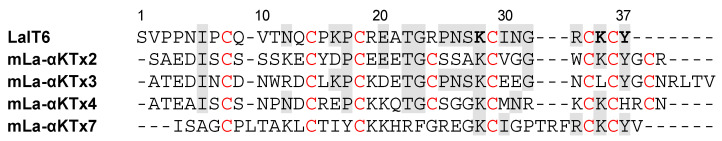
Comparison of the sequence of LaIT6 with those of other synthesized α-KTx peptides in this study. Residues in bold indicate that they are responsible for the activity. Residues identical to those in LaIT6 are shaded, except for Cys residues shown in red.

When comparing the sequences of the five α-KTx peptides synthesized in this study, it was found that mLa-αKTx2 and mLa-αKTx7 contain three residues that were demonstrated to be responsible for the activity of LaIT6 (Figure 4). However, these peptides do not share a high sequence similarity with LaIT6, except for the three important residues in the C-terminal region and cysteine residues. In addition, mLa-αKTx2 has a fourth disulfide bond between the fourth and eighth cysteine residues, while LaIT6 only has three. Although mLa-αKTx7 has three disulfide bonds, it has a long sequence between the fourth and fifth cysteine residues. These differences may explain why the α-KTx peptides other than LaIT6 did not show insecticidal activity.

**Figure 5:**
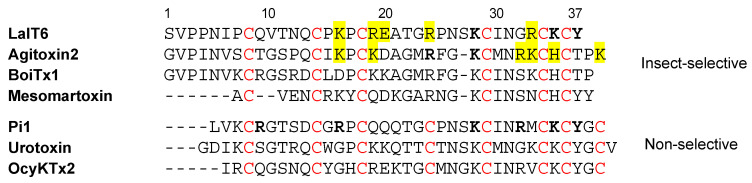
Comparison of the sequence of LaIT6 with those of insect-selective and non-selective α-KTx peptides. Residues in bold indicate that they are important for activity, while those shaded in yellow indicate that they are not important for activity. Cys residues are shown in red.

Although there have been only a few reports of α-KTx peptides exhibiting insecticidal activity, many α-KTx peptides are known to act on insect Shaker K^+^ channels. Besides, several α-KTx peptides, such as agitoxin 2, BoiTx1, and mesomartoxin [26,27,28], have been shown to selectively act on insect Shaker K^+^ channels (Figure 5). When comparing their sequences to that of LaIT6, it is observed that only one of the two lysine residues responsible for the activity of LaIT6 exists in those peptides. This residue (K28 in LaIT6, K27 in agitoxin 2 and BoiTx1, and K19 in mesomartoxin) is highly conserved in α-KTx peptides, and the activity is significantly decreased by its substitution with alanine in both LaIT6 and agitoxin 2, indicating that its importance for binding to K^+^ channels. Substitution of K35 in LaIT6, which is not present in agitoxin 2, BoiTx1, and mesomartoxin, maintained its insecticidal activity to some extent (Table 3), suggesting that K35 plays an additional role in the activity of LaIT6. In agitoxin 2, the arginine residue (R24) in its central region was also reported to be important for activity [29]. However, substitution of the corresponding arginine residue (R24) in LaIT6 did not affect the activity in the present study. When comparing the spatial position of these residues in each predicted or reported 3D structure, R24 in agitoxin 2 faces the same direction as K27, but R24 in LaIT6 faces a different direction from K28 (Figure 2). This suggests that the contribution of R24 in LaIT6 to the interaction with K^+^ channels differs from that in agitoxin 2. The spatial positions of these two basic residues (R24 and K27) in BoiTx1 are similar to those of agitoxin 2, but those in mesomartoxin (R16 and K19) have positions similar to those of LaIT6. This difference is probably due to the absence of a C-terminal tyrosine residue in agitoxin 2 and BoiTx1. In BoiTx1 and agitoxin 2, the phenylalanine residue (F25) is located in a position spatially similar to that of Y37 in LaIT6 (Figure 2), suggesting that this phenylalanine residue is involved in the functional dyad of agitoxin 2 and BoiTx1. 

On the other hand, little is known about the structural factors that contribute to the highly selective action of agitoxin 2, BoiTx1, and mesomartoxin on the insect Shaker K^+^ channel. Pi1 is one of the α-KTx peptides that exhibit inhibitory activity against the K^+^ channels of both insects (Shaker B) and mammals (rat Kv1.2) [22]. In Pi1, the four basic residues (R5, R12, R28, and K31) that constitute the basic ring have been shown to be important in the interaction with rat Kv1.2 channels, in addition to the functional dyad (K24 and Y33) in the C-terminal region (Figure 5) [30]. However, other non-selective α-KTx peptides, urotoxin and OcyKTx2, do not have basic residues corresponding to them in the N-terminal region [31,32]. In addition, the substitution of basic residues (K16, R19, R24, and R33) other than those involved in the functional dyad in LaIT6 resulted in little or no reduction in activity (Table 3). Therefore, it is possible that non-basic residues in LaIT6 contribute to its high selectivity for insect K^+^ channels. 

**Figure 6:**
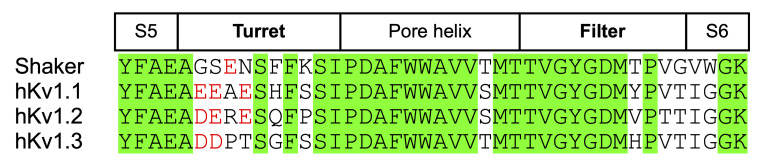
Comparison of the sequence of pore regions of the Shaker and Kv1.x channels. Identical residues are shaded in green. Acidic residues in the turret region are shown in red.

α-KTx peptides are known to bind to the outer vestibule of K^+^ channels, and their interaction with turret and filter regions is particularly important [12]. Furthermore, it has been revealed that the action selectivity of several α-KTx peptides for hKv1.1, hKv1.2, and hKv1.3 channels is due to differences in the sequences of these regions [26,33]. There are also clear differences in the sequence of the turret and filter regions between Shaker and hKv1.x channels. As shown in Figure 6, there are two or three acidic residues on the turret region of the hKv1.x channels, whereas only one acidic residue exists in the Shaker channel, and its position is also different. In addition, the C-terminal side of the turret region in the Shaker channel contains residues (Phe and Lys) with different properties from those in the hKv1.x channels. The sequence at the C-terminal side of the filter region is also slightly different between Shaker and hKv1.x channels (Thr in Shaker; Tyr, Val, and His in hKv1.x). These differences between Shaker and hKv1.x channels may be involved in the action selectivity of LaIT6. In particular, the acidic residues in the turret region can interact with the basic residues of α-KTx peptides through electrostatic interactions. Considering that only one acidic residue exists in a different position in the turret region of the Shaker channel, the electrostatic interaction between this region and the basic residues of the α-KTx peptide is unlikely to be significantly involved. This is supported by the fact that basic residues other than those in the functional dyad of LaIT6 are not important for insecticidal activity. As mentioned above, the non-basic residues of LaIT6 may be responsible for interacting with the turret and filter regions of the Shaker channel through hydrogen bonding and/or hydrophobic interactions. In this regard, the three-dimensional structures of the Shaker and Kv1.2 channels have recently been elucidated using cryo-electron microscopy [34,35]. Based on information obtained from structural biology and structure-activity relationship studies, it will be possible to analyze detailed interactions between the K^+^ channel and LaIT6 in the complex, which will advance our understanding of the structural factors underlying its action selectivity.

## 4. Materials and Methods

### 4.1. Materials

Venom from *L. australasiae* was obtained as previously described [36]. All resins and coupling reagents used for peptide synthesis were purchased from commercial suppliers: Rink amide ProTide (LL) resin, Cl-MPA ProTide resin, and Oxyma Pure (CEM, Charlotte, NC, USA); Rink amide AM resin (Novabiochem, Billerica, MA, USA); 1-[bis(dimethylamino)methylene]-1*H*-1,2,3-triazolo [4,5-b]pyridinium 3-oxide hexafluorophosphate (HATU) (Tokyo Chemical Industry, Tokyo, Japan); Fmoc-NH-SAL-PEG resin and 1-hydroxybenzotriazole (HOBt) (Watanabe Chemical Industries, Hiroshima, Japan), *N*,*N*′-diisopropylcarbodiimide (DIC) (FUJIFILM Wako Pure Chemicals, Osaka, Japan). All other chemicals were commercially obtained.

### 4.2. Peptide Synthesis

Peptides were synthesized using either the manual or the automatic method as previously described [18]. The following solid-phase resins were used for the synthesis of each peptide: Rink amide ProTide (LL) resin (0.19 mmol/g) for mLa-αKTx1 (LaIT6), its analogs, and mLa-αKTx4; Fmoc-NH-SAL-PEG resin (0.22 mmol/g) for mLa-αKTx2; and Cl-MPA ProTide resin (0.17 mmol/g) for mLa-αKTx3 and mLa-αKTx7. In brief, the following conditions were employed. In manual synthesis, deprotection of the Fmoc group was carried out using 20% piperidine in *N*,*N*-dimethylformamide (DMF) under microwave irradiation (Initiator+, Biotage, Uppsala, Sweden) at 80 °C. Each Fmoc-protected amino acid was introduced into the resin in the presence of HATU and *N*,*N*-diisopropylethylamine (DIEA) in DMF under microwave irradiation at 75 °C. For the introduction of Cys, DIC and HOBt were used, and the reaction was performed under microwave irradiation at 50 °C. When synthesis was performed using the automated synthesizer (Liberty Light, CEM), deprotection of the Fmoc group was performed using 20% piperidine in DMF under microwave irradiation at 75 °C and 90 °C. Each Fmoc-protected amino acid was introduced into the resin in the presence of Oxyma Pure, DIC, and DIEA under microwave irradiation at 75 °C and 90 °C. For the introduction of His, the reaction was performed under microwave irradiation at 25 °C and 50 °C. Cleavage of peptides from the resin and removal of side-chain protecting groups were performed using a trifluoroacetic acid (TFA) solution containing 2.5% H_2_O, 2.5% 1,2-ethandithiol, and 1.0% triisopropylsilane. The peptides precipitated by adding cold diethyl ether were washed with cold diethyl ether and dried in vacuo.

### 4.3. Folding Reaction

Peptide folding was performed as previously described [18]. In brief, peptides in reduced form were dissolved at a concentration of 0.1 mg/mL in 200 mM Tris–HCl buffer (pH 8.5) containing reduced and oxidized glutathione (1 mM and 0.1 mM, respectively). For LaIT6 analogs, a solution of peptides in reduced form (20 mg/mL in 0.1% formic acid) was added to the reaction solution in 10 divided portions at 60 min intervals to achieve a final concentration of 1 mg/mL. The mixture was incubated overnight at 25 °C, and the correctly folded peptide was purified using a C18 column (InertSustain C18, 250 × 14 mm, GL Sciences, Tokyo, Japan). The column was eluted with 0.1% TFA in water and 0.1% TFA in acetonitrile at a flow rate of 5 mL/min using a 40 min linear gradient from 5%–50% of acetonitrile at 40 °C. The results of the LC/MS analysis of purified peptides are shown in Figure S1.

### 4.4. Mass Spectrometry

For the analysis of venom and comparison between natural and synthetic peptides, an Orbitrap Exploris 240 mass spectrometer (Thermo Fisher Scientific, Waltham, MA, USA) equipped with a heated ESI source was used. Detection was performed in the positive mode at a resolution of 60,000. HPLC separation was carried out using a C18 column (Vydac Everest C18, 1.0 × 250 mm, Hichrom, Reading, UK). The column was eluted with 0.1% formic acid in water and 0.1% formic acid in acetonitrile at a flow rate of 50 µL/min using a 45 min linear gradient of 5%–50% of acetonitrile at 40 °C. For analysis of the synthetic peptides, an LCMS-2020 mass spectrometer (Shimadzu, Kyoto, Japan) equipped with an electrospray ion source was used. Detection was performed in the positive mode. HPLC separation was carried out using a C18 column (COSMOCORE 2.6C18, 2.1 × 75 mm, Nacalai Tesque, Kyoto, Japan). The column was eluted with 0.1% formic acid in water and 0.1% formic acid in acetonitrile at a flow rate of 0.3 mL/min using a 35 min linear gradient of 5%–40% acetonitrile at 40 °C. To analyze the purity of the final products, except for mLa-αKTx4, HPLC separation was carried out using a C18 column (DAISOPAK SP-100-3-ODS-P, 2.0 × 150 mm, OSAKA SODA, Osaka, Japan). The column was eluted with 0.1% formic acid in water and 0.1% formic acid in acetonitrile/2-propanol (1:1, v/v) at a flow rate of 0.2 mL/min using a 35 min linear gradient of 5%–40% acetonitrile/2-propanol at 60 °C. To analyze the purity of mLa-αKTx4, HPLC separation was carried out using a C18 column (COSMOSIL 3PBr 2.0 × 150 mm, Nacalai Tesque). The column was eluted with 0.1% formic acid in water and 0.1% formic acid in acetonitrile at a flow rate of 0.2 mL/min using a 35 min linear gradient of 2%–40% acetonitrile at 40 °C.

### 4.5. Bioassay

The insecticidal activity was evaluated as previously described [18]. In this study, we used crickets (*Acheta domesticus*, 45–55 mg), houseflies (*Musca domestica*, 13–17 mg), silkworms (*Bombyx mori*, 225–275 mg), and cockroaches (*Periplaneta americana*, 75–225 mg). Each peptide in distilled water was injected into the abdominal region of insects. As a negative control, distilled water was used. Ten insects were used for each dose, except for doses of 3.1 and 6.3 nmol in LaIT6(R24A), for which 15 and 20 insects were used, respectively. The number of dead insects was counted 48 h after injection (24 h after injection in the case of houseflies). The LD_50_ and its 95% confidence interval (CI) were determined by fitting a sigmoidal dose–response curve (variable slope) using PRISM statistical software (Ver. 4.0, GraphPad Software, La Jolla, CA, USA). Toxicity to mammals was evaluated as previously described [18]. The sample solution in a PBS buffer was injected intracerebroventricularly into mice (male Slc:ICR strain, 20 g) after anesthetizing with isoflurane. As a negative control, PBS buffer was used. Three animals were used for each measurement, and toxic symptoms were monitored for up to 24 h. The experimental protocol was approved by the Ethical Committee for the Welfare of Animals at Kyoto University.

### 4.6. Prediction of Mature Structure

SignalP 5.0 Server [37] was used to predict signal peptide regions. Propeptide regions and C-terminal amidation were predicted by comparing the sequences to those of similar peptides found in public databases.

### 4.7. Electrophysiological Recordings

Electrophysiological experiments were carried out in *Xenopus laevis* oocytes. Linearized plasmids encoding Shaker-IR, hKv1.1, and hKv1.2 channels were transcribed using the T7 mMESSAGE mMACHINE transcription kit (Ambion, Austin, TX, USA). Mature female animals were purchased from Nasco (Fort Atkinson, WI, USA) and were housed in the Aquatic Facility (KU Leuven) in compliance with the regulations of the European Union (EU) concerning the welfare of laboratory animals as declared in Directive 2010/63/EU. The use of *X. laevis* oocytes was approved by the Animal Ethics Committee of the KU Leuven with license number P186/2019. Stage V–VI oocytes were collected from anesthetized female *X. laevis* frogs as previously described [38], with the frogs anesthetized by placement in 0.1% tricaine solution (amino benzoic acid ethyl ester; Merck, Rahway, NJ, USA). Oocyte microinjection was performed using a microinjector (World Precision Instruments, Sarasota, FL, USA), with a programmed cRNA injection volume of 4–10 nL, depending on the channel subtype. The oocytes were incubated in an MBS solution (88 mM NaCl, 1 mM KCl, 2.5 mM NaHCO_3_, 1 mM MgSO_4_, and 5 mM HEPES, pH 7.8) supplemented with 50 mg/L gentamicin sulfate and 90 mg/L theophylline. Electrophysiological measurements were performed at room temperature (18–22 °C) using the two-electrode voltage clamp (TEVC) technique. Data were obtained using a GeneClamp 500 amplifier (Axon Instruments, Burlingame, CA, USA), and Clampex9 software (Axon Instruments, Union City, CA, USA) was used for data acquisition and storage. Glass micropipettes were produced using glass capillaries (borosilicate WPI 1B120-6) and drawn in a WPI (World Precision Instruments, Sarasota, FL, USA) manual stretcher. The bath and perfusion solutions were ND96 (96 mM NaCl, 2 mM KCl, 1.8 mM CaCl_2_, 2 mM MgCl_2_, and 5 mM HEPES, pH 7.4). Whole-cell currents of oocytes were recorded 1 to 3 days after injection. Current and voltage electrodes were filled with 3 M KCl, and their resistance was adjusted from 0.5 to 1.5 MΩ. Currents were sampled at 2 kHz and filtered using a four-pole Bessel low-pass Bessel filter at 0.5 kHz. Leak subtraction was performed using a -P/4 protocol. Potassium currents were evoked from a holding potential of −90 mV by 500 ms depolarizations to 0 mV, followed by a 500 ms pulse to either −50 or −90 mV. The IC_50_ and its 95% CI were determined with *n* ≥ 3 at the tested concentrations by fitting a sigmoidal dose–response curve (variable slope) using PRISM statistical software (Ver. 4.0, GraphPad Software).

### 4.8. Three-Dimensional Structure Prediction

Three-dimensional structures of peptides were modeled using AlphaFold 3 [25].

## 5. Conclusions

In this study, we identified LaIT6, a novel insect-selective α-KTx peptide from *Liocheles australasiae* venom. LaIT6 selectively inhibits insect K^+^ channels without affecting human K^+^ channels, making it a promising candidate for the development of bioinsecticides. These findings also contribute to a deeper understanding of the molecular determinants underlying the ion channel selectivity of α-KTx peptides. Future studies focusing on structural characterization of the complex of LaIT6 and K^+^ channels will further clarify the mechanism of its selective action.

## Figures and Tables

**Table 1:** Precursor and estimated mature sequences of α-KTx peptides deduced from de novo transcriptome analysis of the venom gland of *L. australasiae*.

Precursor	Sequence ^1^	Monoisotopic Molecular Mass of Predicted Mature Structure	
		Calcd.	Obsd. ^2^
La-αKTx1	MNAKFIYILLLTAVMFALYEA SVPPNIPCQVTNQCPKPCREATGRPNSKCINGRCKCY G	4053.88	4053.88
La-αKTx2	MNTKFVFLLLVISTLMPTFDASAEDISCSSSKECYDPCEEETGCSSAKCVGGWCKCYGCRG	4150.53	4150.53
La-αKTx3	MNRNFVFLLLLIVTLMPMLDAATEDINCDNWRDCLKPCKDETGCPNSKCEEGNCLCYGCNRLTV	4803.93	4803.92
La-αKTx4	MNKKFIFLLLVVTTLMPMFDAATEAISCSNPNDCREPCKKQTGCSGGKCMNRKCKCHRCNG	4250.78	4250.77
La-αKTx5	MNAKLVCIVLLTAVMFAPDEASLPPIRIPCYVSKDCRKPCLYLTGTPRSKCINRRCKCYG	4423.23	ND
La-αKTx6	MNKPFCAIFLVVLIMFAVSVLPAESTGGCPVDSLCKSYCKSNKFGTEGKCDGTSCKCAIG	3482.49	ND
La-αKTx7	MNKLACYILICVMVSCLFKVPVAEGISAGCPLTAKLCTIYCKKHRFGREGKCIGPTRFRCKCYV	4400.19	4400.18
La-αKTx8	MRLVIILLLMTTLVLAVGAPLGGAKCSSSTQCTRPCRYAGGTHGKCMNGRCRCYG	3772.62	ND
La-αKTx9	MELKYLLVLLAVTCLVSCQDNSLLPSGSCSRTGICMESCAPFLYQPKYHRRCPAGYVCCTLIY	5024.25	ND

^1^ Mature sequence regions are shown in red, and the amidated C-terminal residue is underlined. ^2^ ND; Not detected.

**Table 2:** Insecticidal activity of α-KTx peptides identified in *L. australasiae* venom against crickets.

Peptide	Structure ^1^	LD_50_ (nmol/g) ^2^
mLa-αKTx1 (LaIT6)	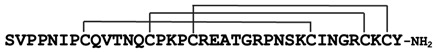	16 (13–19)
mLa-αKTx2	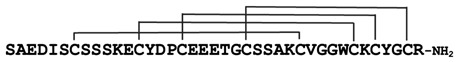	>100
mLa-αKTx3	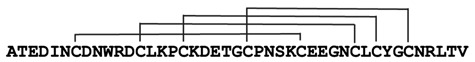	>100
mLa-αKTx4	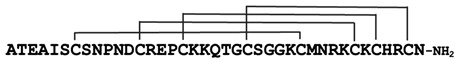	>100
mLa-αKTx7	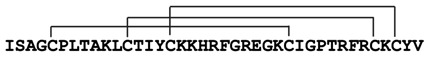	>100

^1^ Disulfide bonding patterns shown in each structure were estimated based on similarities with known α-KTx peptides. ^2^ Values in brackets represent the 95% confidence intervals of the LD_50_.

**Table 3:** Insecticidal activity of LaIT6 and its analogs against crickets.

Peptide	Sequence ^1^	LD_50_ (nmol/g) ^2^
LaIT6	SVPPNIPCQVTNQCPKPCREATGRPNSKCINGRCKCY $‐NH_{2}$	16 (13–19)
LaIT6(K16A)	SVPPNIPCQVTNQCP**A**PCREATGRPNSKCINGRCKCY $‐NH_{2}$	13 (12–16)
LaIT6(R19A)	SVPPNIPCQVTNQCPKPC**A**EATGRPNSKCINGRCKCY $‐NH_{2}$	14 (13–15)
LaIT6(E20A)	SVPPNIPCQVTNQCPKPCR**A**ATGRPNSKCINGRCKCY $‐NH_{2}$	25 (25)
LaIT6(R24A)	SVPPNIPCQVTNQCPKPCREATG**A**PNSKCINGRCKCY $‐NH_{2}$	20 (12–36)
LaIT6(K28A)	SVPPNIPCQVTNQCPKPCREATGRPNS**A**CINGRCKCY $‐NH_{2}$	>100
LaIT6(R33A)	SVPPNIPCQVTNQCPKPCREATGRPNSKCING**A**CKCY $‐NH_{2}$	12 (4–33)
LaIT6(K35A)	SVPPNIPCQVTNQCPKPCREATGRPNSKCINGRC**A**CY $‐NH_{2}$	95 (50–180)
LaIT6(Y37A)	SVPPNIPCQVTNQCPKPCREATGRPNSKCINGRCKC**A** $‐NH_{2}$	>100
LaIT6(COOH)	SVPPNIPCQVTNQCPKPCREATGRPNSKCINGRCKCY ‐COOH	19 (14–25)

^1^ Substituted Ala residues are shown in bold. ^2^ Values in brackets represent the 95% confidence intervals of the LD_50_.

## Data Availability

The original contributions presented in this study are included in the article/Supplementary Material. Further inquiries can be directed to the corresponding author.

## Supplementary Materials

The following supporting information can be downloaded at https://www.mdpi.com/article/10.3390/molecules30163346/s1, Figure S1: LC/MS analysis of the synthesized α-KTx peptides after HPLC purification; Figure S2: Comparison of LC/MS retention times between synthetic and venom-derived peptides; Figure S3: LC/MS analysis of the synthesized LaIT6 analogs after HPLC purification; and Figure S4: Confidence metrics of the LaIT6 structure predicted using AlphaFold 3.

## Abbreviations

The following abbreviations are used in this manuscript:

NaTx	toxin acting on voltage-gated Na^+^ channels
KTx	toxin acting on voltage-gated K^+^ channels
CS α/β	cystine-stabilized α-helix and β-sheet
HATU	1-[bis(dimethylamino)methylene]-1*H*-1,2,3-triazolo [4,5-b]pyridinium 3-oxide hexafluorophosphate
HOBt	1-hydroxybenzotriazole
DIC	*N*,*N*′-diisopropylcarbodiimide
Fmoc	9-fluorenylmethyloxycarbonyl
SPPS	solid phase peptide synthesis
DMF	*N*,*N*-dimethylformamide
DIEA	*N*,*N*-diisopropylethylamine
TFA	trifluoroacetic acid
LD_50_	dose required to induce death in half of animals
IC_50_	half maximal inhibitory concentration
CI	confidence interval
