# Supplementary material for: LaIT6: A Novel Insect-Selective K+-Channel Toxin from Liocheles australasiae Scorpion Venom
Source: Molecules. 2025 Aug 11;30(16):3346. doi: 10.3390/molecules30163346 (PMC12542817; doi:10.3390/molecules30163346)
Supplement: Supplementary file 1 [file molecules-30-03346-s001.zip › molecules-3751040-supplementary.pdf]

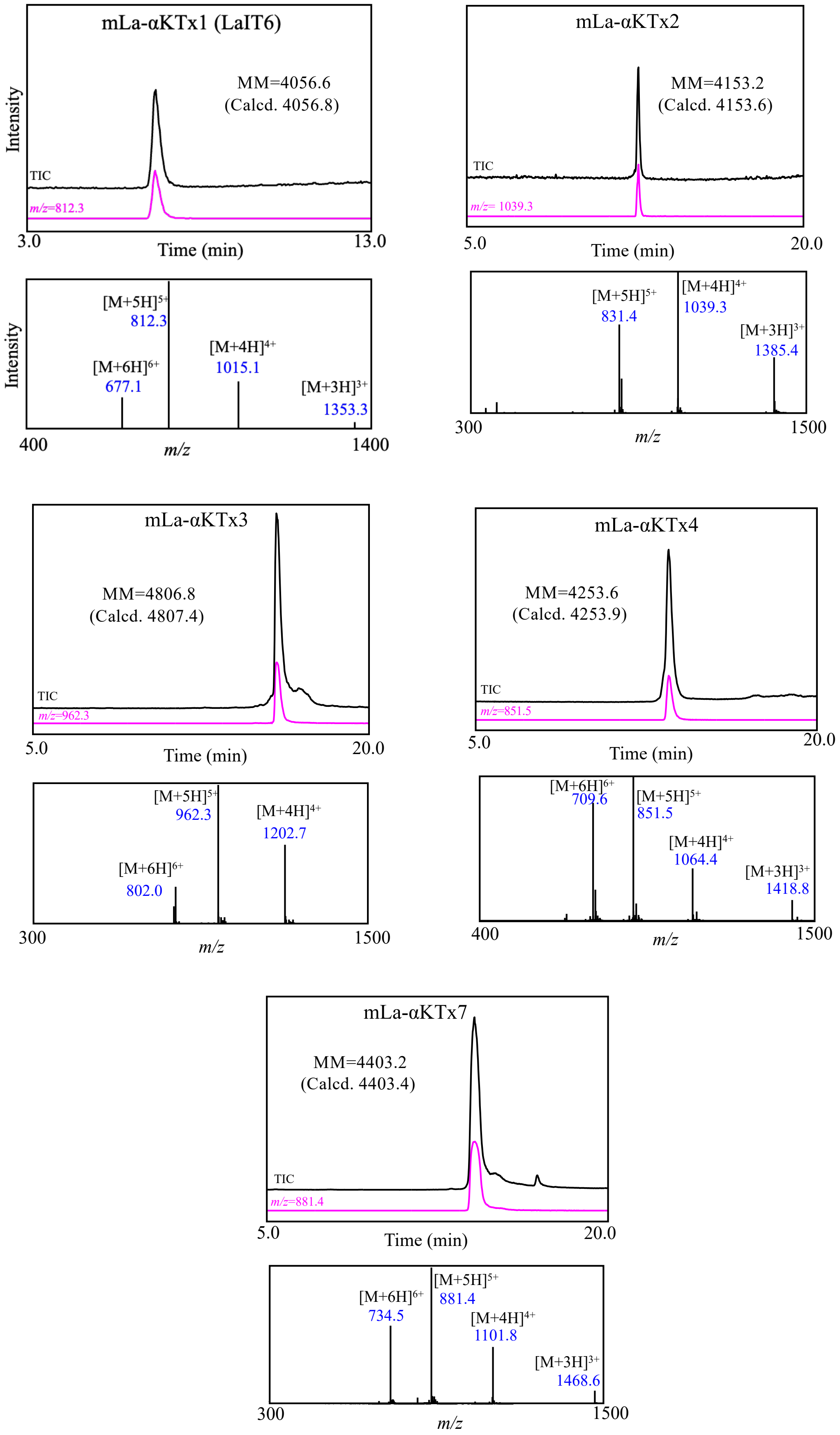

Figure S1 LC/MS analysis of the synthesized α-KTx peptides after HPLC purification.

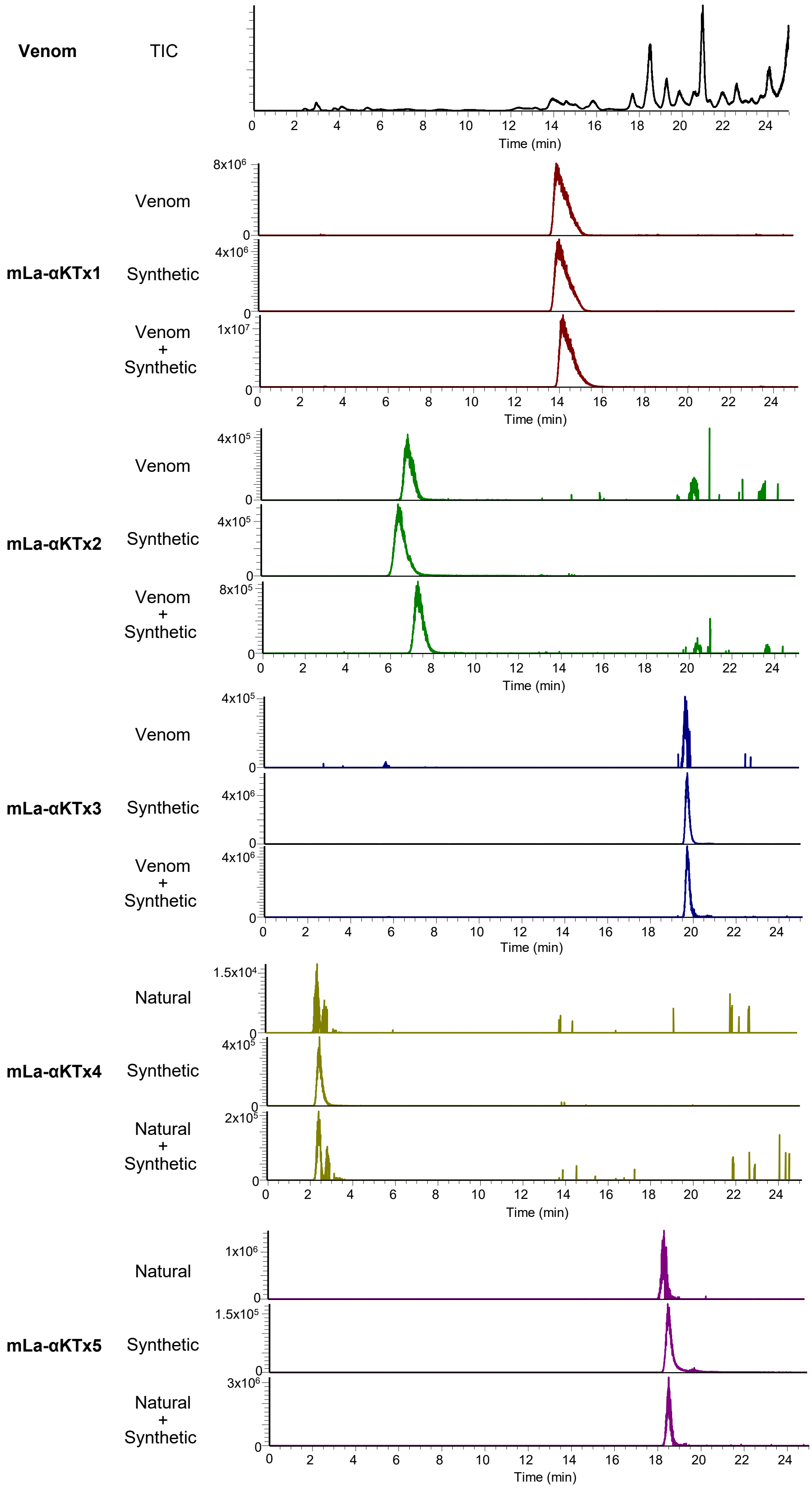

Figure S2 Comparison of LC/MS retention times between synthetic and venom-derived peptides

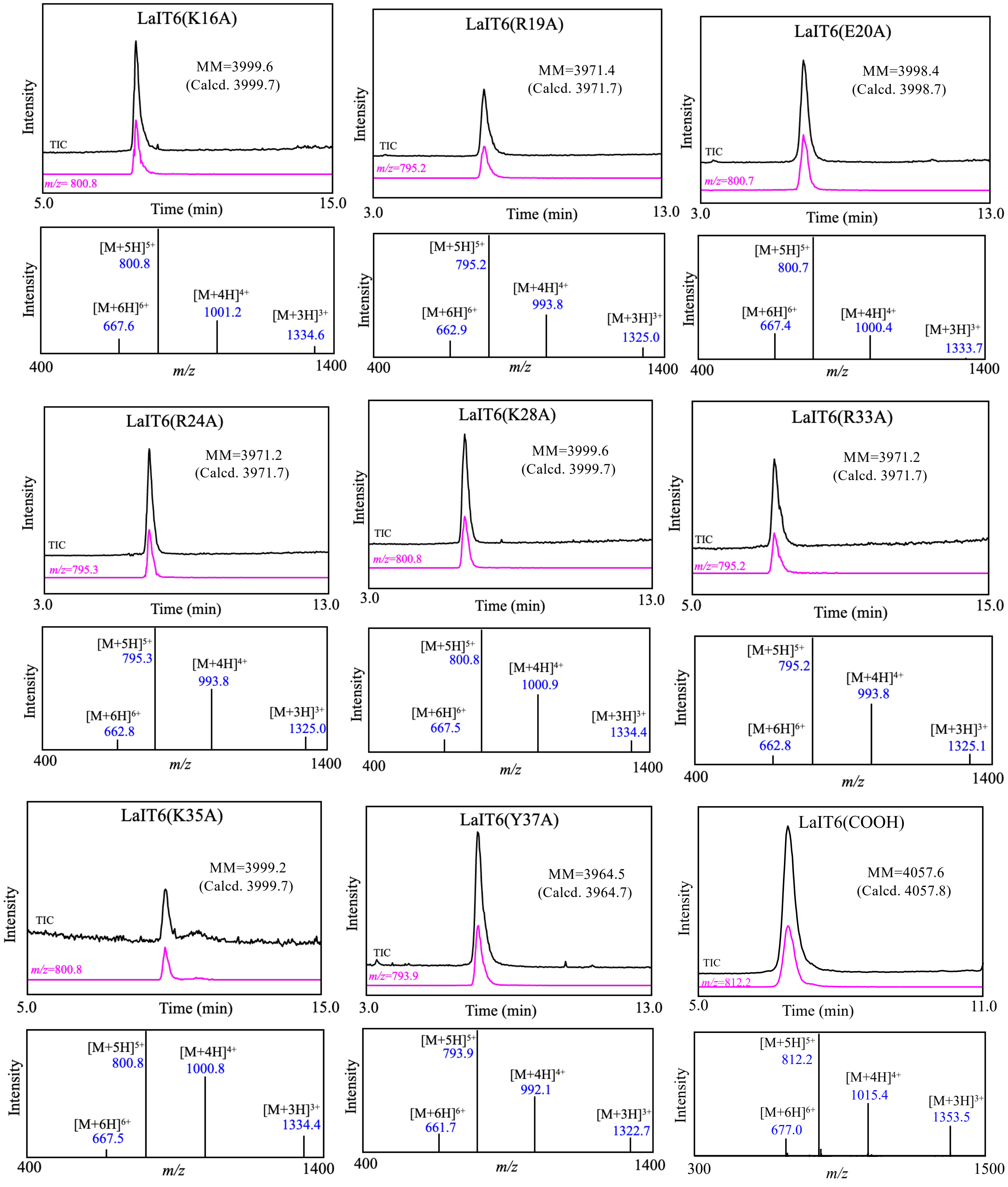

Figure S3 LC/MS analysis of the synthesized LaIT6 analogs after HPLC purification.

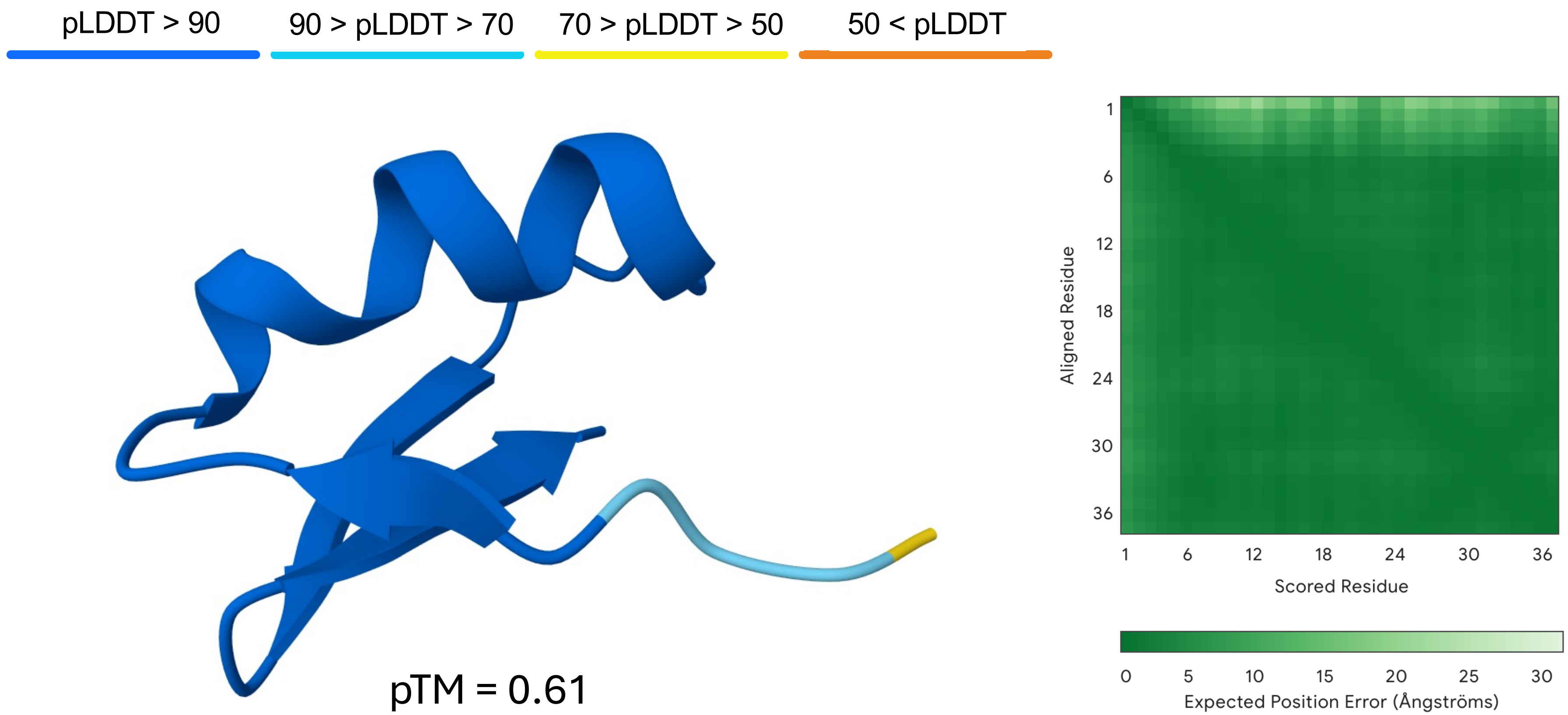

Figure S4 Confidence metrics of the LaIT6 structure predicted using AlphaFold 3.
